# Cross-Domain Person Re-Identification Based on Multi-Branch Pose-Guided Occlusion Generation

**DOI:** 10.3390/s25020473

**Published:** 2025-01-15

**Authors:** Pengnan Liu, Yanchen Wang, Yunlong Li, Deqiang Cheng, Feixiang Xu

**Affiliations:** School of Information and Control Engineering, China University of Mining and Technology, Xuzhou 221116, China; lb22060005@cumt.edu.cn (P.L.); wyc_wl@cumt.edu.cn (Y.W.); ts20060083a31@cumt.edu.cn (Y.L.); xufx92@cumt.edu.cn (F.X.)

**Keywords:** cross-domain, person re-identification, pose-guided occlusion, multi-branch

## Abstract

Aiming at the problems caused by a lack of feature matching due to occlusion and fixed model parameters in cross-domain person re-identification, a method based on multi-branch pose-guided occlusion generation is proposed. This method can effectively improve the accuracy of person matching and enable identity matching even when pedestrian features are misaligned. Firstly, a novel pose-guided occlusion generation module is designed to enhance the model’s ability to extract discriminative features from non-occluded areas. Occlusion data are generated to simulate occluded person images. This improves the model’s learning ability and addresses the issue of misidentifying occlusion samples. Secondly, a multi-branch feature fusion structure is constructed. By fusing different feature information from the global and occlusion branches, the diversity of features is enriched. This enrichment improves the model’s generalization. Finally, a dynamic convolution kernel is constructed to calculate the similarity between images. This approach achieves effective point-to-point matching and resolves the problem of fixed model parameters. Experimental results indicate that, compared to current mainstream algorithms, this method shows significant advantages in the first hit rate (Rank-1), mean average precision (mAP), and generalization performance. In the MSMT17→DukeMTMC-reID dataset, after re-ranking (Rerank) and time-tift (Tlift) for the two indicators on Market1501, the mAP and Rank-1 reached 80.5%, 84.3%, 81.9%, and 93.1%. Additionally, the algorithm achieved 51.6% and 41.3% on DukeMTMC-reID→Occluded-Duke, demonstrating good recognition performance on the occlusion dataset.

## 1. Introduction

Person re-identification is a technology to search whether the same pedestrian exists in the cross-angle camera. The person re-identification is a basic component of intelligent monitoring systems, and is widely regarded as a sub-problem of image retrieval [1,2].

Before the emergence of deep learning, early studies on person re-identification relied on traditional methods. These methods depended on manually designed features and effective measurement functions for identification and matching. Traditional person re-identification algorithms were studied extensively and demonstrated good performance in certain situations [3]. However, the features extracted by these algorithms were often not sufficiently recognizable and contained limited information. With the development of deep learning, significant progress has been made in the field of computer vision. Research on person re-identification based on deep learning has attracted extensive attention from both industry and academia [4].

Currently, supervised learning for person re-identification based on deep learning has advanced significantly, and many effective supervised learning algorithms have emerged [5]. However, this success depends on datasets with a large number of pedestrian labels. The goal of person re-identification is to effectively match identities in cross-domain situations. Due to large differences in feature distribution, supervised algorithm models that perform well on source domain datasets often show poor generalization on cross-domain datasets. To address these issues, researchers are focusing on unsupervised person re-identification methods that have weak label dependence. Two popular research directions in unsupervised person re-identification exist: unsupervised learning [6,7] and Unsupervised Domain Adaptation (UDA) [8,9,10,11,12]. Many researchers are currently engaged in the study of UDA.

In the field of domain adaptation, the categories of research objects in the source and target domain datasets are the same or similar. For example, animals in different datasets exhibit highly similar characteristics. This approach mainly aims to solve the problem of sample differences between domains. However, person re-identification differs from this type of research. In person re-identification, each individual in the two domain datasets has an independent ID. Moreover, individual differences between persons are significant. Conventional domain adaptation methods are less effective in cross-domain person re-identification tasks. Therefore, new methods have been adopted by scholars for the study of Unsupervised Domain Adaptation in cross-domain person re-identification. Based on GAN, Qu et al. [13] developed an attribute-aware style adaptation method called AA-CamStyle, which builds on CamStyle to integrate fine-grained style adaptation with discriminative person re-identification. However, the GAN approach often introduces noise when generating occluded pedestrian images, resulting in a decline in image quality that subsequently impacts re-identification tasks. Wu et al. [14] proposed the IDGAN network, which effectively transfers identity features of different pedestrians’ images to various poses, viewpoints, and background structures by utilizing semantic maps. This significantly increases the diversity and scale of the training data. However, using random vectors or pose skeletons as generative guidance information fails to provide prior knowledge about body contours and identity-related features, resulting in generated images having issues such as inaccurate features, distortions, and blurriness. In addition, adding GAN to the network significantly increases the number of parameters. The generalization of such methods, without knowledge transfer, is insufficient in cross-domain scenarios.

Furthermore, in the context of person re-identification, the phenomenon of occlusion significantly exacerbates the challenges associated with feature extraction and identity verification. Occlusion leads to the loss of certain features of the pedestrian, thereby impeding the model’s ability to capture comprehensive identity information. Additionally, occlusion may induce substantial variations in appearance, further diminishing the accuracy of the recognition process [15]. To deal with problems caused by occlusion, Wang et al. [16] proposed a transformer-based Pose-guided Feature Disentangling method, using pose information to clearly disentangle semantic components and selectively match non-occluded parts correspondingly. Dong et al. [17] generated adversarial representations with incomplete information, misaligned information, and noisy information by erasing, transforming, and adding noise to feature maps to simulate the issues of missing information, position misalignment, and noise caused by occlusion, with the purpose of protecting the re-ID system from various occlusion perturbations. Zhao et al. [18] proposed an Auto-Occlusion Controller (AOC) module that generates independent occlusion policies for each training image based on the semantic features extracted from the pedestrian re-identification network. This module is optimized during the training process using online reinforcement learning methods. And the approach enhances the robustness of the model by effectively simulating occlusion scenarios during training.

In summary, to ensure the invariability of image identity and improve the model’s generalization, this paper proposes a multi-branch pose-guided occlusion cross-domain person re-identification method. The proposed algorithm effectively uses pose points for data augmentation and dynamically calculates similarity between individuals through cross-convolution. This method adapts well to unknown new scenes. The main contributions of the proposed algorithm are as follows:

(1) A pose-guided occlusion generation (POGO) module is proposed. This module extracts information about person pose and position. It enhances data using this information to effectively simulate real-world occlusion without changing individual identities.
(2) A multi-branch mining of person identification features is proposed. The extraction of more complete global and key local information is proposed. Features from different branches are superimposed to enrich identification information, thus improving the model’s generalization ability.
(3) A cross-convolution module is used to cross-convolve the feature map from multi-branch fusion with the feature map in memory. It generates convolution kernels based on the feature maps for matching, calculates similarity between images, and addresses the issue of fixed model parameters.
(4) The Rank-1 and mAP values of the proposed algorithm on MSMT17 Market 1501 and MSMT17 DukeMTMC-reID reached 83.0%, 74.5%, 59.2%, and 60.0%. These results demonstrate the effectiveness of the method.

## 2. Network Framework for Multi-Branch Pose Guided Occlusion Generation

**Figure 1:**
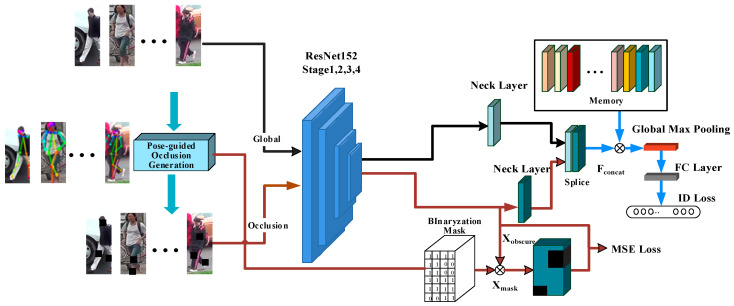
The network framework proposed in this paper.

As shown in Figure 1, this paper designs a new network framework. It consists of three parts: the backbone network structure, the global branch, and the occlusion branch. The POGO module in the network is inspired by a learning strategy that progresses from easy to difficult. This module does not directly generate occluded areas on the image for data enhancement. Instead, it obtains person pose points through pose estimation. As the network’s learning ability is enhanced, the dynamic adjustment of person pose points effectively simulates occluded images in real situations. The network gradually adapts to occluded images during the training process.

### 2.1. Pose-Guided Occlusion Generation Module

Data enhancement techniques, such as image cropping and occlusion, improve model robustness and generalization. However, their effectiveness on challenging training samples is limited. To better simulate occluded images and implement a progressively challenging learning strategy, this paper introduces the POGO module. The POGO module utilizes key point guidance to generate occlusion points, gradually increasing their number throughout training. As training progresses, the network’s learning ability strengthens. This module encourages the network to focus on identifying features in visible areas, resulting in improved recognition rates for images with fewer prominent features. Mature pose extraction technology is employed, with lightweight OpenPose [19] selected as the framework for extracting person pose points.

**Figure 2:**
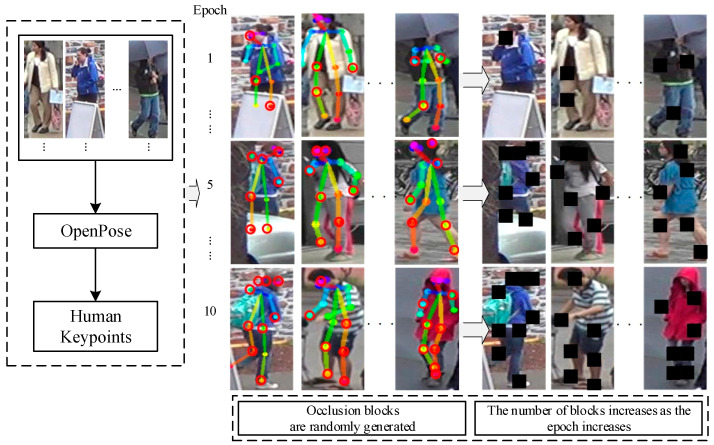
The pose-guided occlusion generation module.

Given an image $I\in R^{3\times H\times W}$, the coordinates of each pose point of the person in the image are obtained in the network and stored in the pose array $pose_{xy}$. According to the increase of the training epoch, the coordinates of i points are dynamically obtained and saved in the occlusion pose array ${occluded\;}_{xy}^{pose}$ in an incremental manner. According to the information of the occlusion pose array, i occlusion areas block $O_{i}$ are generated on the image I, and the area of each $O_{i}$ is $S_{i}$. The H and W are the height and width of the image, and the i and $O_{i}$ are the amount and index of area blocks. Inspired by the learning strategy from easy to difficult, the length of the occlusion pose array ${occluded\;}_{xy}^{pose}$ obtained from the pose array is gradually increased with the increase of the number of training iterations. The occlusion generation process of each training is shown in Figure 2, and the specific steps are as follows:

(1) Assume that the image is sent to the network to extract n pose point coordinates of persons and store them in $pose_{xy}$, where $pose_{xy}$$=\{(x_{1},y_{1}),$$(x_{2},y_{2}),$$\cdots ,(x_{n},y_{n})\}$.
(2) Set the occlusion area of n nodes, and randomly generate i values between $\left[1,n\right]$ by random generation function. Take these i random values as the index of $pose_{xy}$, the corresponding index value is extracted successively and saved to the occlusion node array ${occluded\;}_{xy}^{pose}$$=\{(x_{1},y_{1}),$$(x_{2},y_{2}),$$\cdots ,(x_{i},$$y_{i})\}$.
(3) Take the coordinate points of each joint of ${occluded\;}_{xy}^{pose}$ as the center, set the length h and width w of the occlusion area block. And set the area $O_{i}=$[(max(0,$x_{j}-$$\frac{h}{2})$:min(384,$x_{j}$$+\frac{h}{2})$,max(0,$y_{j}-$$\frac{w}{2}):$min(128,$y_{j}+$$\frac{w}{2})]$. A random value of range $\left[0,\;255\right]$ is generated randomly for the pixels in the specified area, and an artificial occlusion feature tensor is generated to simulate the occluded image $I_{obscure}$ by $S_{i}$.
(4) According to the increase of training epoch, gradually increase the number i of occluded areas.

### 2.2. Feature Extraction

After the input image is processed with special strategies, the POGO module outputs images with different occlusion degrees in the output training stage. The backbone network is divided into two branches, global branch and occluded branch. The global branch extracts the global features with complete image information directly through the backbone network. The local features in the non-occluded region are extracted after the occluded branch is input to the backbone network with a specific data enhancement strategy. The network concatenates the feature information of the two branches according to the channel dimension to achieve the purpose of extracting significant features at multiple levels, suppressing occlusion effects and aligning local features of the body [20].

The global branch will be used to extract the significant region of the image, output the recognizable feature map $X_{A}\in R^{1024\times 24\times 8}$, and $X_{A}$ obtains the feature map $F_{all}\in R^{32\times 24\times 8}$ through the neck layer. On the one hand, this branch extracts the global features of the image, and on the other hand reduces the network parameters [21]. Formula (1) is as follows.

(1) Fall=ReLU(WGXA+bG), Fall∈R32×24×8

where $W_{G}$ and $b_{G}$ are the weight and bias of the convolution layer, respectively.

The occlusion branch extracts local significant features from the image. A feature map $X_{obscure}$ is output that represents local recognition. And $X_{obscure}$ obtains the feature map $F_{obscure}\in R^{32\times 24\times 8}$ through the neck layer. On the one hand, this branch can increase the feature richness, and on the other hand, it can cope well with the problem of image recognition caused by occlusion, Formula (2) is as follows.

(2) $$F_{obscure}=\mathrm{ReLU}(W_{o}X_{obscure}+b_{O}),\;F_{obscure}\in R^{32\times 24\times 8}$$

where, $W_{O}$ and $b_{O}$ are the weight and bias of the convolution layer, respectively.

After extracting the global feature map and occlusion feature map, the global feature map $F_{all}$ and occlusion feature map $F_{obscure}$ are combined according to the channel dimension, and finally the feature map $F_{concat}\in R^{64\times 24\times 8}$ is obtained.

**Figure 3:**
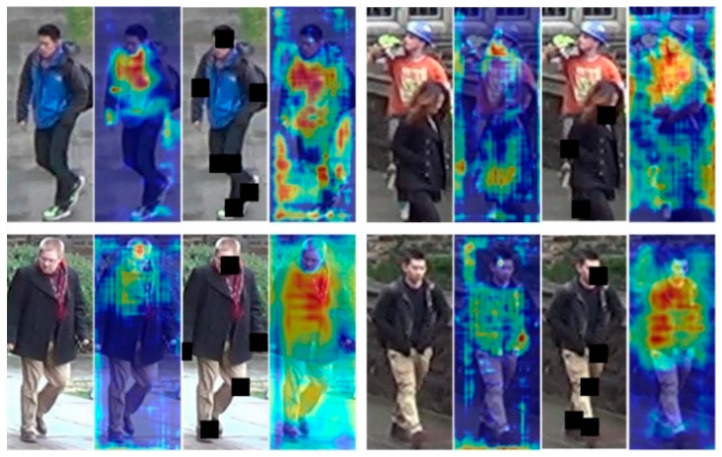
Comparison of heatmaps for introducing the POGO module.

To demonstrate that POGO module effectively improves the extraction and attention to pedestrian features, we visualized the heatmaps of the framework we proposed before and after the addition of the POGO module. The Gradient-weighted Class Activation Mapping (Grad-CAM) is utilized to generate these heatmaps, which highlight the regions of the input image most relevant to the model’s predictions [22]. Figure 3 illustrates the effectiveness of POGO module through heatmaps. From left to right, each group of pictures are: original image, heatmap without POGO, occlusion generated by POGO, heatmap with POGO.

As can be seen from the figure, in different scenes and angles, detection without the POGO module usually only focuses on a small number of pedestrian features, such as faces or joints. Therefore, when faced with new domains or different environments, it can lead to unsatisfactory results because key features are obscured. The introduction of the POGO module can make the algorithm pay attention to more pedestrian features besides face and joints, such as the trunk or clothing. Based on the attention of the proposed architecture to the characteristics of people, it can enhance the re-identification ability of pedestrians in different environments. The introduction of occlusion makes the model better adapt to real objects with different viewing angles and occlusions. Thus, detection performance is improved in real world applications.

### 2.3. Cross-Convolutional Module

The feature map $F\in R^{1\times d\times h\times w}$ is obtained by extracting features from the query image I through the backbone network, where d is the number of output channels, and h and w are the height and width of the feature map, respectively. F is cross-convolved with the feature graph $r_{i}$ in memory to find their matching scores.

**Figure 4:**
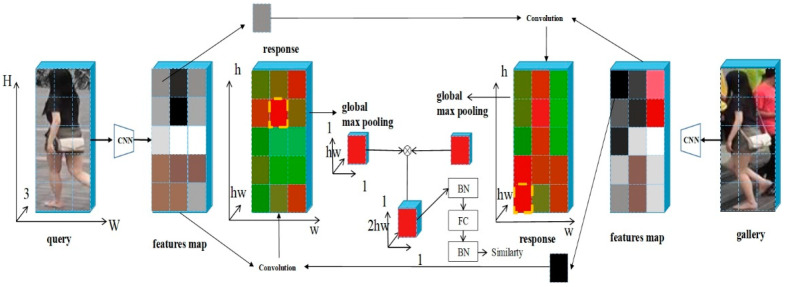
Computation procedure of cross-convolution.

Cross-convolution enhances the contrast of image features by combining information from different channels, making the model more effective in distinguishing similar individuals. Additionally, it reduces computational complexity through weight sharing and local connections, which contributes to the model’s efficiency during both training and inference [23,24]. The detailed cross-convolution calculation process is shown in Figure 4, and the specific calculation steps are as follows:

(1) The feature graph is first normalized according to the channel dimension of the feature graph using L2 paradigm. Local blocks with size [1,1] are traversed at every position in the query feature graph and reorganized into [*hw*,*d*,*s*,*s*] as the convolution kernel.
(2) A dynamic convolution kernel parameter is obtained through step 1, which is no longer fixed to the convolution kernel parameter from the previous training model. Then, the generated convolution kernel is used for convolution calculation in another feature graph. And the cosine similarity of each position in the two feature graphs is calculated, thus generating the similarity score $response\in R^{1\times hw\times h\times w}$. The similarity map can well reflect the local matching results response between two images.
(3) In order to obtain better matching results, the maximum pooling layer is added, and response obtains the corresponding optimal local matching through the maximum pooling operation. Finally, the similarity score is evaluated by sigmoid function, and the loss calculation is carried out in the training stage or the evaluation is carried out in the test stage.

### 2.4. Loss Function

Occlusion branch output feature figure $X_{obscure}$. mask is obtained through binary dimensionality reduction. $X_{obscure}$ and mask are multiplied element-wise to obtain $X_{mask}$. As inputs to the MSE loss, $X_{mask}$ and $X_{obscure}$ are supervised by the MSE loss to ensure that the values of the generated occlusion areas approach 0 as much as possible. The purpose of using MSE loss is to measure the average squared difference between the predicted outputs and the true labels. By minimizing this loss, we aim to reduce the discrepancies between the model’s predictions and the actual data, which helps the model learn more accurate representations of the input features. MSE loss is sensitive to large errors due to the squaring of differences. This characteristic allows the model to focus on significant deviations, which is particularly beneficial in scenarios where accurate predictions are critical. In addition, the MSE loss facilitates smoother convergence during training, enabling the model to adaptively adjust its parameters to improve performance. This allows the model to ignore the generated occlusion area $O_{i}$ during backpropagation. Formula (3) is as follows:

(3) $$L_{MSE}=\mathrm{MSE}(X_{mask},X_{obscure})$$

During training, a fully connected layer (FC) is utilized to treat each pedestrian ID as a separate class, which is crucial for optimizing the loss function to maximize inter-class differences while minimizing intra-class differences. This approach effectively captures the high-dimensional feature distribution necessary for accurate classification. In the identity-aware deep embedding (IDE) framework, each person is assigned a distinct category, with the pedestrian ID serving as the classification label, leading to the classification loss being referred to as ID loss [25]. $F_{concat}$ is cross-convolved with the feature map $F_{memory}$ from the class memory to obtain the local matching result reponse. reponse is integrated into a similarity score through a global max pooling layer and a BN-FC-BN block. The similarity scores between the query image’s feature map and those in the class memory are mapped to the range [0, 1] using the Softmax function. Ultimately, the binary cross-entropy loss is calculated between the query image and the images in the class memory.

The proportion of positive to negative samples in the person re-identification task is severely unbalanced, with the number of negative samples significantly exceeding that of positive samples. To address this issue, focal loss, which is designed to reduce the weight of negative samples during training by focusing more on hard-to-classify examples, is adopted [26]. The loss Formula (4) is as follows:

(4) $$L_{fl}=\left\{\begin{array}{l} -\frac{1}{b}\sum_{i=1}^{b}\sum_{j=1}^{c}\alpha {\left(1-p_{ij}\left(\theta \right)\right)}^{\gamma }\log{\left(p_{ij}\left(\theta \right)\right)}^{\gamma },y_{ij}=1\\ -\frac{1}{b}\sum_{i=1}^{b}\sum_{j=1}^{c}\left(1-\alpha \right){\left(p_{ij}\left(\theta \right)\right)}^{\gamma }\log{\left(1-p_{ij}\left(\theta \right)\right)}^{\gamma },y_{ij}=0\; \end{array}\right.$$

where θ is the network parameter, $y_{ij}=1\;\mathrm{or}\;0$ represents the positive or negative sample, γ and α are the balance parameters, $p_{ij}(\theta )$ is the prediction probability of sigmoid. Focal loss and MSE loss are adopted in the network. The total loss formula is as follows:

(5) $$L=L_{fl}+L_{MSE}$$

## 3. Experiments

### 3.1. Dataset and Evaluation Indicator

Experiments in this section are conducted on four commonly used person re-identification datasets: Market1501 [27], DukeMTMC-Reid [28], MSMT17 [29], and Occluded DukeMTMC [30]. Additionally, experiments are tested directly across domains. During the experiments, the test set is divided into a query set and a gallery set. Candidate images are sorted from largest to smallest based on the matching scores of each query person image against all candidate images.

In the following sections, M, D, M17, and Occluded-Duke will refer to Market1501, DukeMTMC-Reid, MSMT17, and Occluded DukeMTMC, respectively. The person re-identification task utilizes Rank-1 in the cumulative match characteristic curve (CMC [31]) and the mean Average Precision (mAP) [27] as evaluation metrics.

### 3.2. Performance Test

The model is cross-domain evaluated in M→D, D→M, M17→D, and M17→D, and the experimental results are shown in Table 1. In this paper, we utilize ResNet-152 as the baseline model for cross-domain person re-identification tasks. ResNet-152, a deep residual network, is known for its ability to effectively learn hierarchical features through its skip connections, which mitigate the vanishing gradient problem in deep networks [32]. This architecture excels in extracting discriminative features from images. However, one major drawback is its sensitivity to occlusions and misalignments, which can lead to poor feature matching and, consequently, inaccurate identity recognition [33]. Additionally, the fixed parameters of the model can hinder its adaptability to varying conditions encountered in real-world scenarios.

Compared with the baseline test results, the proposed algorithm without Rerank and Tlift has greatly improved, as shown in Table 1. In the D→M transregional performance test, mAP and Rank-1 reached 44.5% and 74.2%, respectively. In M17→D transregional performance test, mAP and Rank-1 reached 60.0% and 74.5%, respectively. In the M17→M transregional performance test, mAP and Rank-1 achieved 59.2% and 83.0%, respectively.

Experiments show that the evaluation results for mAP and Rank-1 are higher when the network model generated by pose-guided occlusion is trained on more complex datasets. This improvement can be attributed to the larger number of occluded images present in these datasets. During the training process, the person image model simulates real scenes more realistically by generating occlusions through pose guidance. As a result, the generalization ability of the network model is enhanced.

**Figure 5:**
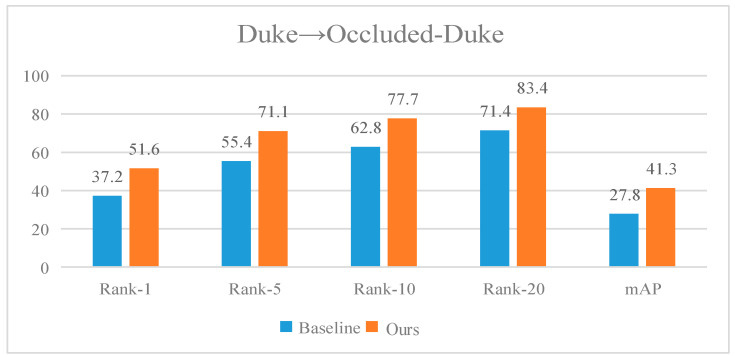
Performance test of the algorithm in this paper in the occlusion dataset.

In addition, to demonstrate that the proposed algorithm can adapt to the problem of decreased detection accuracy due to feature loss from occlusion, tests are conducted based on the D→Occluded-Duke scenario to validate its effectiveness. The experimental results are shown in Figure 5. It is evident that the algorithm presented in this paper achieves the best recognition performance, with Rank-1, Rank-5, Rank-10, Rank-20, and mAP reaching 51.6%, 71.1%, 77.7%, 83.4%, and 41.3%, respectively. Compared to the baseline, the proposed algorithm improves Rank-1 and mAP by 14.4% and 13.5%, respectively. Experiments indicate that the addition of the PGOG module enhances the robustness of the network from simple to complex data enhancements, further demonstrating the positive impact of PGOG on the model.

### 3.3. Ablation Experiment

In the M→D and D→M cross-domain performance tests, ablation experiments of the POGO module are conducted separately. The influence of the POGO module on the learning accuracy of the network is analyzed. In this section, an ablation experiment of the POGO module is conducted. Different occlusion intensities are used to enhance the data, while other settings remain unchanged. Rerank and Tlift are not employed in this comparison. The influence of various occlusion generation strategies on performance is studied, with results shown in Table 2. Occlusion intensity levels are categorized into Level 1 and Level 2. In addition, a random erasing technique for occlusion has been introduced as a comparison.

Firstly, the coordinates of 18 pose points of persons in the image are obtained through Openpose. At different intensity levels, the index value of corresponding number ${\mathrm{num}}_{\mathrm{xy}}$ is randomly generated according to the size of the trained epoch. And ${\mathrm{num}}_{\mathrm{xy}}$ occlusion block is generated according to the coordinate of the pose point. Formula (6) is as follows:

(6) $$level\;1=\left\{\begin{array}{l} num_{xy}=3,epoch\leq 5\\ num_{xy}=6,5\text{<}epoch\leq 10\\ num_{xy}=8,10\text{<}epoch\leq 15\\ num_{xy}=10,15\text{<}epoch\leq 20 \end{array}\right.\;,\;level\;2=\left\{\begin{array}{l} num_{xy}=6,epoch\leq 5\\ num_{xy}=8,5\text{<}epoch\leq 10\\ num_{xy}=10,10\text{<}epoch\leq 15\\ num_{xy}=15,15\text{<}epoch\leq 20 \end{array}\right.$$

The results indicate that the addition of the POGO module improves the model’s ability to recognize occluded images, making the network more robust. Under Level 1 conditions, D→M achieves the best results in both Rank-1 and mAP. Conversely, under Level 2 conditions, M→D yields the best performance in Rank-1 and mAP. Furthermore, the results demonstrate that the high-intensity enhancement method yields superior results on smaller, simpler datasets (M), while the low-intensity enhancement method performs better on larger, more complex datasets (D). This observation aligns with the model’s learning ability, which typically progresses from weak to strong as training epochs increase. In this study, the enhancement coefficient of Level 1 is applied to D→M, while the enhancement coefficient of Level 2 is used for M→D.

To validate the effectiveness of the proposed multi-branch framework, ablation experiments were conducted on each branch, and the results are shown in Table 3. The experiments were conducted on M→D and D→M.

To evaluate the impact of different loss functions on model performance, we conducted ablation experiments. The results of these experiments are summarized in Table 4.

In addition, we conducted an ablation experiment on the cross-convolution module, and the results are shown in Table 5.

By changing the size of the local block, the convolution kernel parameters of different sizes are obtained dynamically. Table 3 shows the trend of algorithm performance change caused by the change of block Size. And Rerank and Tlift are not used in this comparison.

As shown in Table 6, when the local block of the proposed algorithm is set to 1, Rank-1 and mAP of D→M and M→D are both optimal. When the size of the local block increases by an integer multiple k, the convolution kernel size changes to [$\left(h_{0}w_{0}\right)/k^{2},\;d,\;s_{0}k,\;s_{0}k$]. $h_{0}w_{0}$ and $s_{0}$ represent the kernel parameters before the block size change.

Specifically, the smaller the region block is, the size of dynamic convolution kernel will change accordingly. The traversal efficiency of convolution kernels with different sizes directly affects feature sampling and extraction. In addition, the adaptive dynamic convolution kernel with small area blocks leads to efficient traversal, which makes feature sampling and extraction are more effective, thus improving the pedestrian re-identification performance.

### 3.4. Contrast Experiment

To demonstrate the advantages of the framework proposed in this section, the test results of the proposed algorithm are compared with current mainstream algorithms. The performance comparison results on M→D, D→M, M17→D, and M17→M cross-domain datasets are shown in Table 7.

As shown in Table 7, the comparison algorithms include 18 algorithms that use the dataset of the unsupervised domain adaptive, 4 algorithms that use the dataset of the target domain for unsupervised learning, and 6 algorithms that directly perform cross-domain testing.

The method based on the unsupervised domain adaptive is mainly divided into two directions. The first type adopts the dataset of source domain and target domain to train simultaneously to achieve the purpose of reducing the difference between domains. Supervised training is performed on the source domain dataset and unsupervised training is performed on the target domain dataset. For example, in the TSDRC algorithm, both ID knowledge and attributes knowledge are used to train the source domain model, which is supervised by the angular Softmax loss. And for the target domain, TSDRC algorithms generate pseudo labels by the unsupervised clustering algorithm. The second type uses the model pre-trained in the source domain to train in the target domain with unsupervised algorithm, to gradually reduce noise labels. Compared to this type of algorithm, the proposed algorithm in this paper outperforms these algorithms in terms of mAP and Rank-1 in cross-domain tests from D→M, M17→D, and M17→M.

The method based on unsupervised learning mainly uses the unsupervised learning algorithm to extract each image feature, and generates pseudo-labels according to the features with the clustering algorithm to gradually reduce the differences within the class. For example, the HCT method combines hierarchical clustering with hard-batch triplet loss to improve the quality of pseudo labels. However, the pseudo-labels generated when the model learning ability is weak in the early stage have a large amount of noise, which will lead to the underfitting of the model in the later stage. Using dynamic convolution to calculate the similarity between images, the proposed algorithm has obvious advantages on mAP and Rank-1 when tested on the same dataset.

The method based on direct cross-dataset assessment is trained in the source domain dataset, and the evaluation results are obtained directly through cross-domain evaluation.

The method based on direct cross-dataset assessment is trained on the source domain dataset and directly evaluated across domains to obtain assessment results. The method proposed in this paper has advantages over such algorithms, showing improvements in both mAP and Rank-1 metrics. For example, the mAP and Rank-1 of MMT baseline on D→M and M→D is, respectively, 22.6% and 36.8%, 17.1%, and 27.9% less than that of the proposed algorithm.

## 4. Conclusions

In this paper, a multi-branch pose-guided occlusion person re-identification method is proposed. On one hand, PGOG is utilized to gradually block the image. On the other hand, multiple branches are employed to extract various distinguishing features, enhancing the extraction of both global and local features to enrich the representation of individuals with specific identities. Additionally, cross-convolution and dynamic feature matching between images are implemented to address the issue of fixed model parameters.

The algorithm effectively separates background information from foreground information, generating more valuable occlusion images. Dynamic convolution is applied to perform similarity calculations on the fused feature maps, significantly improving the accuracy of the person re-identification model. Cross-domain verification is conducted on the Market1501, DukeMTMC-reID, MSMT17, and Occluded-Duke datasets.

Compared to existing algorithms, this paper makes notable contributions to the performance of cross-domain person re-identification. The proposed network model demonstrates superior performance in real-time computation and generalization capabilities, offering higher practical application value. Furthermore, the model provides better pre-trained models for other transfer algorithms, enhancing model accuracy after transfer learning.

## Figures and Tables

**Table 1:** The network performance results of proposed algorithm.

Method	Training	Rank-1	mAP
Baseline	Market→Duke	54.4	33.6
	Duke→Market	62.8	31.6
	MSMT→Duke	82.2	78.4
	MSMT→Market	88.4	76.0
Ours without Rerank and Tlift	Market→Duke	57.2	38.1
	Duke→Market	74.4	44.5
	MSMT→Duke	74.5	60.0
	MSMT→Market	83.0	59.2
Ours	Market→Duke	71.1	63.2
	Duke→Market	87.9	72.4
	MSMT→Duke	81.9	80.5
	MSMT→Market	93.1	84.3

**Table 2:** Ablation experiments with different occlusion intensity.

Method	Training	Test: Duke		Training	Test: Market	
		Rank-1	mAP		Rank-1	mAP
Baseline	Market	54.4	33.6	Duke	62.8	31.6
Ours (Random Earing)		55.0	34.1		67.3	35.2
Ours (Level 1)		56.7	36.9		74.4	44.5
Ours (Level 2)		57.2	38.1		72.8	43.5

**Table 3:** Ablation results of the multi-branch framework.

Branch	Target: Duke			Target: Market		
	Source	Rank-1	mAP	Source	Rank-1	mAP
Global Branch	Market	55.6	36.6	Duke	72.5	42.8
Occlusion Branch	Market	56.3	37.5	Duke	73.1	43.6
Combined Branch	Market	57.2	38.1	Duke	74.2	44.5

**Table 4:** Ablation results of different loss functions.

Loss Function	Target: Duke			Target: Market		
	Source	Rank-1	mAP	Source	Rank-1	mAP
Focal Loss	Market	57.0	37.9	Duke	73.9	44.1
MSE Loss	Market	56.8	37.8	Duke	73.7	44.2
Focal Loss + MSE Loss	Market	57.2	38.1	Duke	74.2	44.5

**Table 5:** Ablation results of the cross-convolution module.

Module	Target: Duke			Target: Market		
	Source	Rank-1	mAP	Source	Rank-1	mAP
Without cross-convolution module	Market	56.8	37.6	Duke	73.6	44.0
With cross-convolution module	Market	57.2	38.1	Duke	74.2	44.5

**Table 6:** Impact of block size on the detection result.

Local Block Size	Target: Duke			Target: Market		
	Source	Rank-1	mAP	Source	Rank-1	mAP
1 × 1	Market	57.2	38.1	Duke	74.2	44.5
	MSMT	74.5	60.0	MSMT	83.0	59.2
2 × 2	Market	56.8	37.5	Duke	73.7	44.0
	MSMT	74.2	59.5	MSMT	82.7	58.9
3 × 3	Market	56.6	37.4	Duke	73.5	43.9
	MSMT	73.9	59.3	MSMT	82.5	58.8

**Table 7:** Comparison of results of proposed algorithm with other person re-identification algorithms.

Method	Target: Duke			Target: Market		
	Source	Rank-1	mAP	Source	Rank-1	mAP
SPGAN [34]	Market	46.9	26.4	Duke	58.1	26.9
DTGAN [35]	Market	44.9	25.0	Duke	56.7	25.8
CASC [36]	Market	51.5	30.5	Duke	64.7	35.6
	MSMT	59.3	37.8	MSMT	65.4	35.5
ECN [37]	Market	63.3	40.4	Duke	75.1	43.0
PAUL [38]	Market	56.1	35.7	Duke	66.7	36.8
	MSMT	72.0	53.2	MSMT	68.5	40.1
CBN [39]	Market	58.7	38.2	Duke	72.7	43.0
	MSMT	66.2	46.7	MSMT	72.8	42.9
UCDA [40]	Market	55.4	36.7	Duke	64.3	34.5
CDS [41]	Market	67.2	42.7	Duke	71.6	39.9
MAR [42]	MSMT	43.1	48.0	MSMT	67.7	40.0
HUDA [43]	Market	68.5	37.6	Duke	52.3	30.2
SPA [44]	Market	65.2	52.4	Duke	73.9	53.4
DDAF [45]	Market	70.1	49.1	Duke	84.5	63.0
NECA [46]	Market	57.0	34.7	Duke	65.5	34.8
DFEF [47]	Market	56.9	34.4	Duke	68.3	36.3
IPES-GAN [48]	Market	55.7	33.3	Duke	66.8	34.4
SILC [49]	Market	68.5	50.3	Duke	80.7	61.8
TSDRC [50]	Market	70.3	44.3	Duke	73.5	41.2
NIN [51]	Market	79.4	62.6	Duke	86.6	68.7
BUC [52]	Duke	47.4	27.5	Market	61.0	30.6
SSL [53]	Duke	52.5	28.6	Market	71.7	37.8
ACAN [54]	Duke	67.6	45.1	Market	73.3	50.6
HCT [7]	Duke	69.6	50.7	Market	80.0	56.4
ACDBNet [55]	-	-	-	MSMT	79.9	50.7
MMT baseline [56]	Market	54.0	35.4	Duke	65.3	35.6
PAUL baseline [38]	MSMT	65.7	45.6	MSMT	59.3	31.0
OSNet-AIN [57]	MSMT	71.1	52.7	MSMT	70.1	43.3
DTIN-Net [58]	Market	57.0	36.1	Duke	69.8	37.4
CI3 [59]	Market	67.6	48.7	Duke	73.7	46.0
	MSMT	70.3	54.6	MSMT	73.7	48.1
Ours	Market	71.1	63.2	Duke	87.9	72.4
	MSMT	81.9	80.5	MSMT	93.1	84.3

## Data Availability

The data presented in this study are available from the corresponding author upon request.
